# Coherent pipeline for biomarker discovery using mass spectrometry and bioinformatics

**DOI:** 10.1186/1471-2105-11-437

**Published:** 2010-08-26

**Authors:** Ali Al-Shahib, Raju Misra, Nadia Ahmod, Min Fang, Haroun Shah, Saheer Gharbia

**Affiliations:** 1 Health Protection Agency, Centre for Infections, 61 Colindale Avenue, London, NW9 5EQ, UK

## Abstract

**Background:**

Robust biomarkers are needed to improve microbial identification and diagnostics. Proteomics methods based on mass spectrometry can be used for the discovery of novel biomarkers through their high sensitivity and specificity. However, there has been a lack of a coherent pipeline connecting biomarker discovery with established approaches for evaluation and validation. We propose such a pipeline that uses *in silico *methods for refined biomarker discovery and confirmation.

**Results:**

The pipeline has four main stages: Sample preparation, mass spectrometry analysis, database searching and biomarker validation. Using the pathogen *Clostridium botulinum *as a model, we show that the robustness of candidate biomarkers increases with each stage of the pipeline. This is enhanced by the concordance shown between various database search algorithms for peptide identification. Further validation was done by focusing on the peptides that are unique to *C. botulinum *strains and absent in phylogenetically related *Clostridium *species. From a list of 143 peptides, 8 candidate biomarkers were reliably identified as conserved across *C. botulinum *strains. To avoid discarding other unique peptides, a confidence scale has been implemented in the pipeline giving priority to unique peptides that are identified by a union of algorithms.

**Conclusions:**

This study demonstrates that implementing a coherent pipeline which includes intensive bioinformatics validation steps is vital for discovery of robust biomarkers. It also emphasises the importance of proteomics based methods in biomarker discovery.

## Background

Within the last decade, mass spectrometry has been widely used for identifying and characterising proteins within complex mixtures. This is mainly due to its high sensitivity, specificity, mass accuracy and good dynamic range. Some of the identified proteins can be characterised as unique to the organism thus qualifying them as potential biomarkers. Such biomarkers can be used to monitor the pathological changes within cells and lead to a better understanding of disease processes while improving diagnostics. Furthermore, they can also be used to detect biological agents thus providing a fast and accurate identification system [1,2]

In a typical bottom up mass spectrometry proteomic approach, a complex protein mixture is first digested with a proteolytic enzyme such as trypsin. The resulting peptides are separated by liquid chromatography and subjected to analysis by mass spectrometry. The peptides are then ionized (in MS mode) and selected ions are further fragmented (in MS/MS mode). The resulting MS/MS spectra displaying the ion fragments derived from the selected peptide ions are submitted to search algorithms for peptide identification [3]. The most efficient method to identify the amino acid sequence from the MS/MS spectra is based on searching the spectra against a protein sequence database. This is done using various algorithms such as Sequest [4], Mascot [5] and X!Tandem [6]. Generally, all the algorithms follow the same principle: since peptide fragmentation can be predicted, the algorithms compare an observed MS/MS fragmentation pattern with theoretical (predicted) fragmentation patterns from peptides (in proteins) contained in the selected protein database. The output will be a list of peptide sequences that have the closest match to the observed spectrum.

Database search algorithms apply various constrained search parameters when implementing comparisons. These include the mass tolerance, proteolytic enzyme constraints (only peptides that conform to the digestion rules of the proteolytic enzyme are included), number of missed cleavage sites and amino acid modifications. However, algorithms differ in their implementation of the scoring system when comparing the fragment ion spectrum against the theoretical fragmentation patterns in the database. This score, which can be more than one for a single algorithm, ultimately differentiates between the true and false peptide identifications (i.e. the likelihood that the peptide identified has occurred by chance). Typically those peptides that have a score higher than the threshold (assigned manually by the user) are accepted and presented.

The popular Sequest algorithm applies the spectral correlation functions scoring system where it calculates the cross correlation score (Xcorr) for each fragment ion spectrum for all the candidate peptides searched in the database. Mascot on the other hand applies a probability-based score instead of a score reporting the number of matched peaks. Finally, X!Tandem uses a statistical scoring system for its peptide assignment as it converts the database search score into an expectation value which provides an estimate of whether or not the observed match is random.

**Figure 1:**
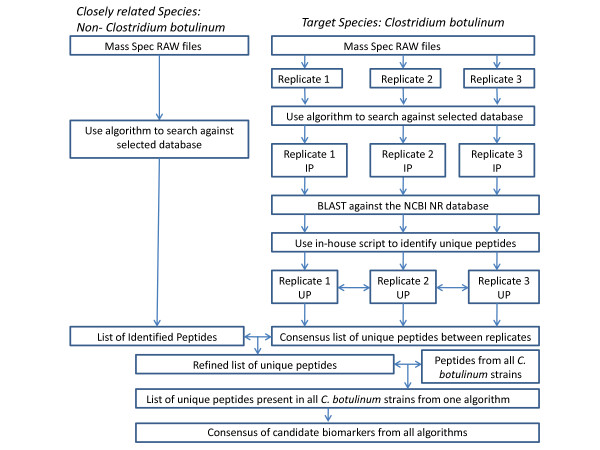
**Proteomics pipeline for biomarker discovery**. IP is Identified Peptides and UP is Unique Peptides. This process is applied to each search algorithm and the refined list of markers from each algorithm is compared and a consensus of the peptides in all algorithms is selected for a final validated list of markers.

Discovering biomarkers in proteomics has been done using database search algorithms [7] mass spectral peak finding [8] and binning [9]. In this study we go beyond that and evaluate the peptides identified by the database search algorithms using intensive bioinformatics validation methods. To achieve this we have implemented a pipeline that identifies peptide biomarkers based on mass spectrometry and *in silico *data analysis (Figure 1). The *in silico *approaches involve selecting candidate markers from BLAST [10], verifying the candidates by comparison with a control, and further validating the markers by using a consensus approach of database search algorithms. Studies have shown that accuracy in protein identification increases by using a concordance of database search algorithm scores [11-14]. Together with increasing peptide coverage and thus biomarker coverage, there are two other reasons for applying this approach in our pipeline. First, as the algorithm scores differ between each algorithm, the number of peptides identified by each algorithm will be different. Therefore if one was to consider the results from a single algorithm, the likeliness of selecting the 'correct' biomarker will be minimal. Second, as the search databases are often incomplete and the correct match may not be available, the MS/MS spectrum may be matched to an incorrect peptide sequence thus resulting in false positive identification. Two search engines can provide very different results from the same MS/MS spectrum [11].

The model bacterium used in this study was *Clostridium botulinum*, a lethal pathogen that causes a rare form of food poisoning known as botulism. It can be used as a potential bioterrorism agent because of its high lethality and mode of transmission.

A crucial step in any study that focuses on the discovery of species specific biomarkers is to eliminate markers that are also present in other species. This ensures that any biomarkers discovered are only unique to those strains of interest. *C. botulinum *consists of a collection of phenotypically diverse strains that do not cluster within a single phylogenetically coherent group [15]. Due to these properties, a panel of species that belong to the genus were selected so that the specificity of any potential peptide biomarker can be tested. This pipeline may provide a standard for biomarker discovery using mass spectrometry and provide a useful tool for microbial identification and disease diagnosis. The pipeline is described in detail in Figure 1.

## Results and Discussion

Proteomic analysis provides tools for examining gene products and their features such as protein-protein interactions, post-translational modifications, protein isoforms and subcellular localization. As proteins are the functional component of cells, the rapid and sensitive identification of proteins is essential for organism identification and disease diagnosis. This can be achieved by discovering robust protein markers through the use of tandem mass spectrometry based proteomics which is characterised by its high sensitivity, specificity, mass accuracy and good dynamic range.

Various database search algorithms have been used to identify peptides by searching the MS/MS spectra against a protein sequence database. Each algorithm adapts a particular scoring system for peptide identification. To generate a biomarker discovery system therefore, there is a need to outline a comprehensive workflow of mass spectrometry data analysis that includes intensive validated steps in achieving a robust biomarker list. Here, we have developed such a pipeline. Six *Clostridium botulinum *strains and an additional eight *Clostridium *species that range from species that are phylogenetically closely related to *C. botulinum *(e.g. *Clostridium Sporogenes*) to species that are distantly related (e.g.*Clostridium baratii *) were used to identify robust biomarkers that can delineate *Clostridium botulinum *from any other pathogen.

### Peptide Identification

From each algorithm, the number of peptides identified for each replicate ranged between ± 300. This variation between biological replicates is expected and allows for the comparison between the replicates in the later phase of the pipeline. As shown in Table 1, more peptides were identified by Sequest followed by X!Tandem and Mascot. These results agree with Kapp *et al *[12] who performed a comparison of the algorithms using blood specimen data. It is interesting, however, to note the large difference between Mascot and the other two algorithms. Sequest identified about double the number of peptides identified by Mascot while X!Tandem identified around 2500 more peptides than Mascot. One reason for this could be the fairly stringent Mascot scores applied (Mascot ion score greater than 20).

**Table 1:** Number of peptides identified by each algorithm

Algorithm(s)	Number of peptides from raw files	Number of biomarkers	
X!Tandem	6920	34	
Mascot	4465	23	
Sequest	7923	12	
X!Tandem *∩ *Mascot	2324	17	
X!Tandem *∩ *Sequest	3342	12	
Sequest *∩ *Mascot	2241	9	
X!Tandem *∩ *Sequest *∩ *Mascot	2081	8	

*∩ *indicates the overlap between the algorithms specified.

### Measuring the performance of algorithms

One consequence of the scoring system used by database search algorithms is the possibility for false positive peptide identifications. The peptides returned by each algorithm are not necessarily the result of a perfect match in the database but may be due to coincidental similarity in MS/MS fragmentation patterns [16]. This can lead to a very large number of incorrect peptide sequence assignments in large scale proteomics experiments. One way to measure the portion of incorrect peptide assignments is to apply a target-decoy search strategy [17,18]. This is done by reversing the target proteins of the search database to generate a decoy database. After the algorithm has identified the peptides, the correct (True Positive) and incorrect (False Positive) hits are counted and the false discovery rate (FDR) is calculated by FP/(FP+TP).

Mascot uses the target-decoy strategy and calculates the FDR for each raw spectral file (.RAW file). There, the performance was satisfactory as the overall FDR was around 3%. For Sequest, the FDR had to be calculated manually by concatenating the decoy with the target database [18]. Results show the expected number of incorrectly identified peptides were 988, while the total number of peptides identified was 24939. Thus FP = 988, TP = 23951 and FDR = 988/24939 = 3.9%.

### Unique peptide identification

**Figure 2:**
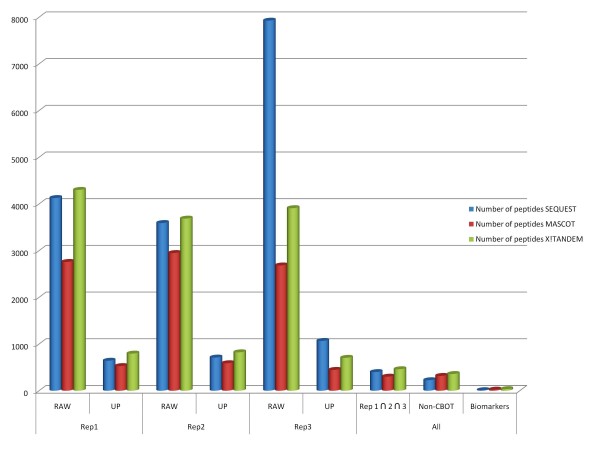
**Refinement of peptides**. Unique Peptides (UP) were identified by selecting the peptides that had an exact hit in BLAST against the NCBI non-redundant database. These peptides were refined by selecting the consensus of peptides in all biological triplicates (Rep *∩ *1 *∩ *2 *∩ *3) followed by selecting the peptides that are not present in non-*Clostridium botulinum *species (Non-CBOT). The final phase selects only those peptides that are present in all six *C. botulinum *strains (All strains). We have labelled these peptides as 'Biomarkers'.

Using WU BLAST [19] and in-house scripts, unique peptides from each replicate of *Clostridium botulinum *were identified. Uniqueness was measured by selecting the peptides that had an exact match with an amino acid sequence in the NCBI non redundant database (Figure 2). The selected peptides equate to 5% of the overall peptides identified by X!Tandem and Mascot and 4% for Sequest. The lists of peptides from each replicate for every algorithm were compared to eliminate the non-conserved peptides amongst the three replicates. For X!Tandem, 43% of the peptides were conserved in all the three replicates, followed by 38% and 30% for Mascot and Sequest respectively.

### Comparison with other Clostridia species

**Figure 3:**
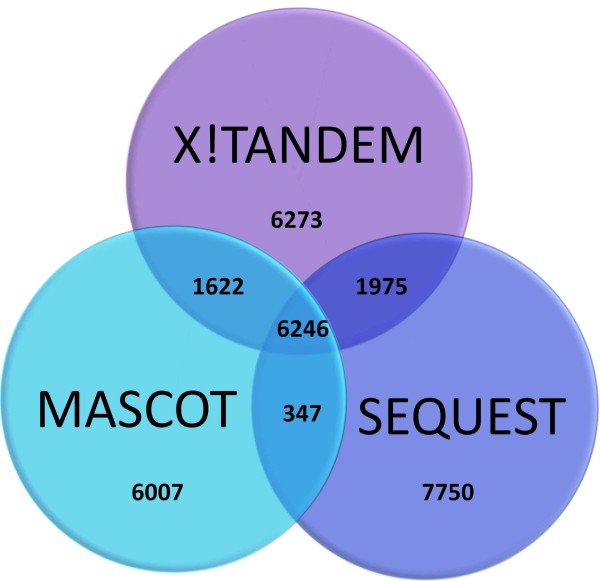
**non-Clostridium botulinum species union**. Venn diagram showing the intersection between all peptides identified from non-*Clostridium botulinum *species by X!Tandem, Mascot and Sequest.

Peptides were identified from eight non-*botulinum *Clostridial species searched against the Swiss-Prot Protein database [20]. For all eight species, we see a similar trend with the *C. botulinum *strains where Sequest identified 16318 peptides followed by X!Tandem with 16116 and finally Mascot with 14222 peptides (Figure 3). This could be because of Mascot's fairly strict score statistic that limits the algorithm for identifying peptides. The non-*Clostridium botulinum *peptides were compared with peptides identified from *Clostridium botulinum *to ensure the peptides identified are only present in *C. botulinum *and not any other *Clostridium *species. Peptides identified by Mascot were refined by 25%, X!Tandem 25% and Sequest 20% which highlight the importance of experimental controls.

To examine whether the unique peptides selected are conserved amongst all *C. botulinum *strains, a comparison of these peptides across all *C. botulinum *strains was performed. For X!Tandem, 357 peptides were present in at least one of *C. botulinum *strains and 33 were conserved across all strains. As for Mascot, 22 unique peptides were conserved from a possible 224 and 11 from a possible 316 were conserved for Sequest.

### Conserved unique peptides

To add further statistical significance to the peptides of interest, we decided to select the peptides that are conserved across the list of unique peptides in all *C. botulinum *strains. This is important for the identification of microorganisms as the biomarkers selected in this study must be present in all *C. botulinum *strains for the detection of the pathogen in an unknown sample. However, this should not be the case for all studies involved in biomarker discovery. In some cases, unique peptides that are present in at least one but not all strains can also qualify as potential biomarkers as it could be specific to certain strains only. It is therefore important to study whether non-conserved peptides should be dismissed prior to the implementation of the pipeline.

### Concordance between database search algorithms

**Figure 4:**
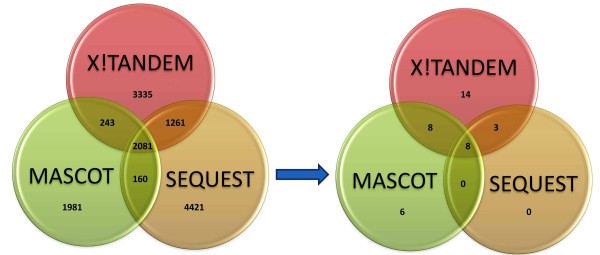
**Overlap and concordance between database search algorithms**. Both diagrams show the intersection between the three algorithms in peptide identification. The diagram on the left shows the intersection of the algorithms at identifying peptides prior to *in silico *validation (first step in the pipeline). The diagram on the right shows the overlap of the different algorithms in biomarker discovery after the complete implementation of the pipeline. The arrow represents the flow of the pipeline to achieve a final refined list of biomarkers.

One possible way of validating the performance of a database search algorithm and to maximise peptide coverage is to have other algorithms corroborate the results from a single algorithm. Here, the refined list of unique peptides from each algorithm were collected and compared to achieve a final list of candidate biomarkers that have been recognised by all algorithms (Figure 4). Some overlap of the result is evident when comparing two algorithms. For example, X!Tandem and Mascot overlap by 8 peptides while Sequest only overlaps by 3 peptides with X!Tandem and none with Mascot. However, the level of overlap seems to increase when a third algorithm is introduced. This achieves a final list of 8 candidate biomarkers that are identified as a consensus of the three algorithms, which ultimately gives higher confidence to each of the peptides identified by all algorithms.

There are three reasons for this. First, each algorithm implements a different scoring system that identifies the peptides according to a particular score. By utilizing the complementary score strength of each algorithm, we can maximise biomarker coverage and achieve higher confidence in the identification. Second, by combining the results from different search engines, we expect a reduction in the noise level obtained from the MS/MS spectra. This is important because the discriminatory effectiveness of the algorithm is reduced by the presence of noise or low quality MS/MS spectra. Thirdly, the search databases are often incomplete which can result in incorrect peptide identification. This is the case as the algorithm will return a match for all the spectra, regardless of whether the correct peptide/protein is present in the database [11]. Therefore, to increase the chances of selecting a robust biomarker, it is good practice to apply a union of multiple algorithms that can reduce the number of incorrect peptide identifications and provide more robust markers.

For these reasons, we have assigned a confidence scale for biomarker identification with algorithm intersection. Table 2 shows the confidence assignment of the candidate biomarkers discovered after the implementation of the pipeline using *C. botulinum *MS/MS data. Highest to lowest confidence (Rank 1-3) was assigned respectively to the unique peptides that were identified by all three algorithms followed by peptides identified by two algorithms and finally peptides that were exclusively identified by a single algorithm. The need for this confidence scale provides the researcher or the clinician with three sets of candidate biomarkers that can be given priorities for further experimental validation. From this, we avoid discarding peptides that can potentially be important signatures in species identification and clinical diagnosis.

**Table 2:** Sequence and functions of C. botulinum candidate biomarkers

Rank	Biomarker Sequence	Algorithm(s)	Protein Annotation	
1	EAEYIFGNFGK	X *∩ *M *∩ *S	Unknown	
	EDLTNVFDLSER	X *∩ *M *∩ *S	Unknown	
	ENLITAPENTTIGEAK	X *∩ *M *∩ *S	Metabolism Multiple pathways	
	GMDTLLESFIK	X *∩ *M *∩ *S	Metabolism Multiple pathways	
	INEFPEILEYK	X *∩ *M *∩ *S	Enzyme Families	
	MNEIMQDDTLER	X *∩ *M *∩ *S	Carbohydrate Metabolism	
	TIVSLDEIEIK	X *∩ *M *∩ *S	Translation	
	YANIADYLSLGGK	X *∩ *M *∩ *S	Unknown	

2	AAGLEIGETGAIK	X *∩ *M	Unknown	
	AFIESVEEALEGGEK	X *∩ *M	Replication and Repair	
	GAYVLNKEEIEK	X *∩ *M	Metabolism Multiple pathways	
	GEPIVLDNGAVAGQAFR	X *∩ *M	Replication and Repair/Cell motility	
	IMNDFSMLASK	X *∩ *M	Unknown	
	NDIVVVSPDLGSVTR	X *∩ *M	Metabolism Multiple pathways	
	QAATAIDEYLSK	X *∩ *M	Metabolism Multiple pathways	
	TSAEGIEIVAK	X *∩ *M	Amino Acid Metabolism	
	DLSTNSWTMIR	X *∩ *S	Replication and Repair	
	IIDMNNAAIDEGVNAIVK	X *∩ *S	Unknown	
	TTTIPSMVEALSR	X *∩ *S	Translation	

3	AMESILVVIPAEK	X	Unknown	
	CLVAEEETGLTTR	X	Metabolism Multiple pathways	
	EALTEYLLNMSTR	X	Metabolism Multiple pathways	
	EGSFIYVIGPK	X	Energy Metabolism	
	GIENVNVFTVR	X	Unknown	
	GLEVQEEVLNK	X	Nucleotide Metabolism	
	GTNIVNIIPIENNEK	X	Replication and Repair	
	GTNIVNLIPIENNEK	X	Replication and Repair	
	IGNIVEHEETPQISGMIK	X	Translation	
	ISTVGDVVDYIK	X	Unknown	
	KWDEDKFEEVMK	X	Unknown	
	TFGVELEDEPSGK	X	Amino Acid Metabolism	
	VGIAHNVTPETVEK	X	Amino Acid Metabolism	
	WYIVDAADKPLGR	X	Translation	
	LGLAEDEAIESK	M	Membrane Transport	
	MIGYDIFENEEAK	M	Carbohydrate Metabolism	
	MIGYDLFENEEAK	M	Carbohydrate Metabolism	
	QVLSFVTEETVVQR	M	Unknown	
	SMPALITAISELNQPR	M	Unknown	
	TRFETNLAVANHLVDK	M	Translation	

X = X!Tandem, M = Mascot, S = Sequest and *∩ *= intersection. The first column indicates the rank of the biomarkers. Rank 1 markers have higher confidence than the other markers due to the concordance of the search algorithms in their identification. The second column shows the amino acid sequence of the candidate biomarker. Column 3 shows the algorithm(s) used to identify the candidate biomarker. Where there is no algorithm intersection, the candidate biomarker was exclusively identified by the single algorithm. The final column indicates the functional classification of the proteins (of the listed peptides) obtained from KEGG [21]. The biomarkers fall into 4 different categories: Metabolism, Genetic Information Processing (Replication and Repair, Cell motility and Translation), Enviromental Information Processing (Membrane Transport) and Unknown.

### Candidate biomarker functions

Candidate biomarkers were classified into functional categories using the KEGG Automatic Annotation Server [21]. Proteins assigned to these categories belong to ancient conserved domains and six broad functional categories exist. The results show that candidate biomarkers fall into 4 different broad functional categories (Table 2). This highlights that the peptides identified belong to a diverse range of proteins and the pipeline is not bias towards any specific functional group. Peptide candidate biomarkers were mainly found to belong to the metabolism and genetic information processing categories. The genetic information-processing category includes genes such as ribosomal proteins, initiation factors and elongation factors. This category contains many genes that encode for proteins that have housekeeping functions essential for the living cell. One such example is a candidate peptide present within the prolyl-tRNA synthetase. This protein is essential in bacteria and expressed when a microbial cell is actively growing. Biomarkers derived from such proteins are crucial in the development of diagnostic assays as they represent markers from proteins that are constitutively expressed independent of growth phase. The method described in this paper relies on the detection of peptides from organisms grown under standardised condition but the discovery of biomarkers that originate from essential proteins allows for less stringent growth conditions to be applied and also allows for the detection of biomarkers directly from environmental and clinical samples.

## Conclusions

There is has been a lack of a comprehensive and sensitive pipeline to discover novel biomarkers for microorganism identification and disease diagnosis. Proteomics methods based on mass spectrometry hold special value for the detection of biomarkers due to their high sensitivity and specificity. In this study, we have designed and implemented a proteomic pipeline that uses mass spectral data together with intensive bioinformatics approaches for biomarker discovery. As the peptides are refined in the pipeline, we show that the robustness of the unique peptides increases with every stage of the pipeline. We conclude that 8 *C. botulinum *candidate biomarkers have been selected with high confidence and 31 others selected with less confidence for species identification. Further optimisation of the pipeline can be implemented by the verification of the biomarkers at the DNA level. This is important as the proteomic approach applied here is dependent on protein expression and the genomic validation of these biomarkers at the DNA level ensures specificity in strains where genome data is not available. We envisage the marriage of proteomics and genomics and how this will develop to be a valuable tool for biomarker discovery.

## Methods

### Sample Preparation

Six *Clostridium botulinum *strains, (*C. botulinum *toxin type A NCTC 7272 and clinical isolate AHO6506,*C. botulinum *toxin type B NCTC 7273, NCTC 3807 and NCTC 751, *C. botulinum *toxin type F NCTC 10281) and eight strains representing pathogenic and non-pathogenic phylogenetically related Clostridial species were cultured and protein extractions performed in triplicate. Non-*Clostridium botulinum *strains included four strains of *Clostridium sporogenes*, two strains of *Clostridium perfringens*, one strain of *Clostridium butyricum *and one strain of *Clostridium tetanii*. *Clostridium botulinum *strains were cultured on *Clostridium botulinum *isolation agar under anaerobic conditions at 37°C for 48 hours while non-*C. botulinum *strains were grown under the same conditions but on fastidious anaerobic agar. From each culture, a starting inoculum was transferred using a loopful of cells in 20 ml of Trypticase-Peptone-Glucose-Yeast Extract Broth. The turbidity of the starting culture was adjusted to give an optical density at 600 nm (OD_600_) of approximately 0.3 to ensure broths contained equivalent cell mass before incubation under anaerobic conditions at 37°C for 24 hours to an OD_600 _of 2-2.7. Liquid cultures were centrifuged at 10000 × g for 10 minutes at 4°C to harvest the cells. Broth supernatant was removed and the cell pellet washed with 1 ml 1× TE buffer (Sigma-Aldrich) containing a 1× protease inhibitor cocktail (Roche). The cells were centrifuged at 10000 × g for 10 minutes at 4°C to re-pellet the cells and the supernatant removed. The wash step was repeated before the cell pellet from each sample was used for protein extraction.

The cell pellet was treated with 1.5 ml of 1× TE buffer containing 6 *μ*g/*μ*l lysozyme (Sigma-aldrich) and a 1× protease inhibitor cocktail (Roche) and incubated for 1 hour at 37°C on a heating block. Cells were collected by centrifugation at 8000 × g for 10 minutes at 4°C and the supernatant removed. The pellet was re-suspended in 100 *μ*l of a solubilisation cocktail (30 mM Tris-Cl pH 8.5, 7 M Urea, 2 M Thiorurea, 4% CHAPS and 70 mM DTT) and solubilisation allowed to occur for 30 minutes at room temperature. The suspension was clarified by centrifugation at 21000 × g for 30 minutes at 21°C. Extracts were filtered through 0.2 *μ*m anopore vectra spin filters (Whatman) by centrifugation at 8000 × g and 4°C and the filtrate transferred to fresh 1.5 ml tubes.

The protein concentration of each preparation was measured using a Bradford assay. Proteins (10 *μ*g) were then separated by one-dimensional SDS-PAGE using NuPAGER^® ^Novex 1.0 mm 4-12% Bis-Tris gels (Invitrogen). Each gel lane was excised into 12 bands that were in-gel digested with trypsin (Promega) for 16 h at 37°C. Peptides were extracted from gel bands with 0.1% TFA.

### Liquid Chromatography and Mass Spectrometry

Peptide digests were analysed using online nano liquid chromatography and tandem mass spectrometry (nano LC-MS/MS) on an Ultimate 3000 Dionex nano/capillary HPLC system (Dionex) coupled to a LTQ Orbitrap mass spectrometer (Thermo Electron). The separations were performed on a nano analytical C18 column (75 *μ*m id × 15 cm, 3 *μ*m) (Dionex) using a 45-minute linear gradient of 5 to 45% solvent B (90% CH_3_CN/0.1% formic acid) versus solvent A (2% CH_3_CN/0.1% formic acid), then to 90% B for an additional 5 minutes. The mass spectrometer was operated in a standard data-dependent mode to automatically switch between MS and MS/MS acquisition. The full survey scan (m/z 440-2000) was acquired in the Orbitrap with a resolution of 60,000 at m/z 400, which was followed by six MS/MS scans in which the most abundant peptide precursor ions detected in the preceding survey scan were dynamically selected and subjected for collision-induced dissociation (CID) in the linear ion trap to generate MS/MS spectra.

### Database searching

MS data were generated in the form of .RAW files (Thermo Finnigan file formats), which contain all the spectra detected from the LC-MS/MS analysis for each sample. Each replicate was examined separately. Three database search algorithms were used: X!Tandem (freeware distributed by Global Proteome Machine Organization), Mascot (Matrix Science, UK) and Sequest Bioworks version 3.3 (Thermo Finnigan, San Jose, CA). The same parameters were applied for all three algorithms for a fair assessment of peptide identification. They were: Enzyme: trypsin; Fixed (or static) Modifications: carbamidomethylation of cystine; Variable Modifications: oxidation of methionine; Missed Cleavage Sites: 2; peptide mass tolerance ± 10 ppm. Specific X!Tandem parameters were: refinement was disabled; maximum valid E-value for reported peptides was 0.1.

An in-house Perl script (Additional file 1) was written to export peptide sequences from Mascot .dat files. Sequences that had the specific Mascot score of > 20 were reported. The protein sequence database used by all algorithms was the Swiss-Prot Protein database [20] containing 498110 non-redundant proteins. For false positive discovery assessment, a decoy database was used. Mascot generates this automatically and uses the results to calculate the false discovery rate. A decoy database was used in Bioworks for Sequest using its .reverse function. We decided to concatenate the decoy and target databases as recommended by Elias *et al *[18]. FDR was then calculated based on the true and false positive identifications.

### Data Analysis

The output from each algorithm was analysed and scripts were used to remove duplicate peptides from each replicate and the data was converted to FASTA. For each replicate, BLASTP [10] in WU-BLAST 2.0 [19] was adjusted for short sequences and used to identify specific markers. The command line argument used to run blastp was -v = 500 -b = 40 -filter = none -e = 5000 -mformat = 2 -matrix = pam30 -nogap -hspmax = 40 -cpus = 8. To identify the biomarkers from the BLAST output, an in-house script written in Perl (Additional file 2) was implemented. The script works as follows: For each alignment, if the description states *Clostridium botulinum*, the script looks for 100% sequence identity to the query sequence. If this is satisfied, then the length of the query sequence is determined and searches for an exact match in the alignment. A search for any match in that block of alignments is then made, and if there are no conflicts, the script will label the match as a biomarker. Peptides identified from each replicate were compared to find the peptides that are conserved amongst all three replicates. To ensure that these peptides are unique to *C. botulinum *and not any other species, a comparison of peptides were made from *C. botulinum *to the closest *Clostridium *species as these are genetically the most likely biological systems to have common sequence similarity. The peptides that were shared with these strains were eliminated and a refined list of markers was produced. In the next phase of the pipeline, for each algorithm, only those unique peptides that are present in six *C. botulinum *strains were selected. Finally the three refined lists of markers from each algorithm were compared and the consensus between the three lists was selected as the final marker list. The *in silico *pipeline is summarised in Figure 1.

## Authors' contributions

AA performed the bioinformatics analysis and prepared the manuscript. RM devised and overlooked the study and checked the manuscript. NA performed the sample preparation and contributed to the manuscript. MF performed the mass spectrometry and contributed to the manuscript. HS and SG supervised the study. All authors have read and approved the final manuscript.

## Supplementary Material

Additional file 1 **Extracting peptide sequences with a user set cutt-off score from Mascot .dat files**. This Perl script parses out MASCOT .dat files and outputs the peptide sequence with a score cut-off set below. Input: Mascot .dat files. Output: Peptide sequences identified by Mascot in FASTA format. Click here for file

Additional file 2 **Unique Peptide Identification from WU BLAST output**. This is script written in Perl and will identify potential biomarkers from a WU BLAST output. Input: Peptide sequences identified from MS/MS experiment and WU BLAST output file. Output: Unique peptides in FASTA format. The script works as follows: For each alignment, if the description states e.g. *Clostridium botulinum*, the script looks for 100% sequence identity to the query sequence. If this is satisfied, then the length of the query sequence is determined and searches for an exact match in the alignment. A search for any match in that block of alignments is then made, and if there are no conflicts, the script will label the match as a biomarker. Click here for file
