# Supplementary material for: Coherent pipeline for biomarker discovery using mass spectrometry and bioinformatics
Source: BMC Bioinformatics. 2010 Aug 26;11:437. doi: 10.1186/1471-2105-11-437 (PMC2939613; doi:10.1186/1471-2105-11-437)
Supplement: Additional file 1 — Extracting peptide sequences with a user set cutt-off score from Mascot .dat files. This Perl script parses out MASCOT .dat files and outputs the peptide sequence with a score cut-off set below. Input: Mascot .dat files. Output: Peptide sequences identified by Mascot in FASTA format. [file 1471-2105-11-437-S1.PDF]

```perl
#!/usr/bin/perl
#Author: Ali Al-Shahib, ali.al-shahib@hpa.org.uk

use warnings;
use strict;


my $MASCOT;
my $mascot;
my $mascot_parsed;
my $mascot_parsed_two;
my $two;
my $one;
my $three;
my $peptide;
my $five;
my $six;
my $score;
my $eight;
my $nine;
my $ten;
my $result;
my @peptide;
my $seen;
my $promptString;
my $defaultValue;
my $user_score;
my $required_score;
my $raw;
my $x = 0;
my $mascot_one;
my $mascot_two;
my $mascot_three;
my @mascots;
my @results;
my @label;
my $label;

      my $fileToRead = <STDIN>;
      chomp($fileToRead);
      open (MASCOT, $fileToRead) or die( "Cannot open file : $!" );

      while ($mascot = <MASCOT>) {
                chomp($mascot);
      if($mascot=~/FILE=/g){
            $mascot_one = $mascot;
      if ($mascot_one=~s/FILE=PATH//g){
            $mascot_two = $mascot_one;
      if ($mascot_two=~s/.RAW//g){
                $mascot_three = $mascot_two;
      }
      }
      }
        elsif($mascot=~s/q[0-9]+_p[0-9]+=[0-9]+,//g){
                $mascot_parsed = $mascot;
        if($mascot_parsed=~s/;".*//g){
                $mascot_parsed_two = $mascot_parsed;
      chomp ($mascot_parsed_two);
```

```
            ($one, $two, $three, $peptide, $five, $six, $score, $eight,
$nine, $ten) = split /,/, $mascot_parsed_two;
            if ($score > SET SCORE CUTT-OFF SCORE HERE){
            push @results, "$peptide";
        }

            }

      }

}
      my %seen = ();
      foreach $result (@results) {
            unless ($seen{$result}) {
                  # if we get here, we have not seen it before
                  $seen{$result} = 1;
                  $x++;
                  print ">X_$mascot_three","_",$x,"\n",$result,"\n";
      }
      }

close (MASCOT);
1;
```
