# Supplementary material for: Coherent pipeline for biomarker discovery using mass spectrometry and bioinformatics
Source: BMC Bioinformatics. 2010 Aug 26;11:437. doi: 10.1186/1471-2105-11-437 (PMC2939613; doi:10.1186/1471-2105-11-437)
Supplement: Additional file 2 — Unique Peptide Identification from WU BLAST output. This is script written in Perl and will identify potential biomarkers from a WU BLAST output. Input: Peptide sequences identified from MS/MS experiment and WU BLAST output file. Output: Unique peptides in FASTA format. The script works as follows: For each alignment, if the description states e.g. Clostridium botulinum, the script looks for 100% sequence identity to the query sequence. If this is satisfied, then the length of the query sequence is determined and searches for an exact match in the alignment. A search for any match in that block of alignments is then made, and if there are no conflicts, the script will label the match as a biomarker. [file 1471-2105-11-437-S2.PDF]

```
#!/usr/bin/perl
#Author: Ali Al-Shahib, ali.al-shahib@hpa.org.uk

use warnings;
use strict;

my $qid = 0;
my $sid = 0;
my $E = 0;
my $N = 0;
my $Sprime = 0;
my $S = 0;
my $alignlen = 0;
my $nident = 0;
my $npos = 0;
my $nmism = 0;
my $pcident = 0;
my $pcpos = 0;
my $qgaps = 0;
my $qgaplen = 0;
my $sgaps = 0;
my $sgaplen = 0;
my $qframe = 0;
my $qstart = 0;
my $qend = 0;
my $sframe = 0;
my $sstart = 0;
my $send = 0;
my $peptide = '';
my $blastline = '';
my $length = 0;
my @results;
my $result;
my $peptide_letter;


      my %peptides_hash = ();

            open (PEPTIDES, "SEQUENCE FILE NAME") ||  die $!;
            while ($peptide = <PEPTIDES>) {
            chomp($peptide);
            if ($peptide=~/>/g){
                  $peptide_letter = substr ($peptide,1);
            }

            $length = length($peptide);
            $peptides_hash{$peptide_letter} = $length;
      }
      close(PEPTIDES);

      my $key;
      my $value;
      my $total_matches=0;

      ### loop through all peptides in peptide file
      while (($key, $value) = each(%peptides_hash)){

             my $failed_sid=0;
```

```
                my $passed_length_and_percentage=0;
            my @perfect_score_proteins =();
            my $x=0;
            open (BLAST_OUTPUT, "BLAST OUTPUT FILE") ||  die $!;

            ## for each peptide in the peptide file
            while ($blastline = <BLAST_OUTPUT>) {
                        chomp($blastline);
                ($qid, $sid, $E, $N, $Sprime, $S, $alignlen, $nident,
$npos, $nmism, $pcident, $pcpos, $qgaps, $qgaplen, $sgaps, $sgaplen,
$qframe, $qstart, $qend, $sframe, $sstart, $send) = split /\t/,
$blastline;
$x++;
                next if ($qid ne $key);

                if(
                    (int($alignlen)==$value)
                     &&
                    ($pcident =~ /100/g)
                   ) {
                            $passed_length_and_percentage=1;
                            push (@perfect_score_proteins,$sid);
                     }

            }
             close(BLAST_OUTPUT);

            if(@perfect_score_proteins < 1) {
                   $failed_sid = 1;
            }

            if(@perfect_score_proteins > 1) {
                                   foreach my $protein_name
(@perfect_score_proteins) {

                                if ($protein_name !~
/Clostridium_botulinum/g) {
                                     $failed_sid = 1;
                                }
                         }
               }

               else {
              if ($perfect_score_proteins[0]){
                        if ($perfect_score_proteins[0] !~
/Clostridium_botulinum/g) {
                                          $failed_sid=1;
                         }
              }
               }

             if($failed_sid==0) {
                                   push @results, "$key";
                        print "$x: $key=passed\n";
                        $total_matches++;
                        print "\n\nTOTAL=$total_matches\n\n";

                }
```

```
        else {
              print "$x: $key=failed\n";

        }

  }

   print "\n\nTOTAL MATCHES = " . $total_matches . "\n\n\n";




  open (MYFILE, ">OUTPUT FILE") ||  die $!;

  my %seen = ();
  foreach $result (@results) {
        unless ($seen{$result}) {
              # if we get here, we have not seen it before
              $seen{$result} = 1;
              print MYFILE $result."\n";
        }
  }

  close MYFILE;

1;
```
